# *BRAF V600E* liquid biopsy-based detection in precision oncology

**DOI:** 10.1515/almed-2025-0110

**Published:** 2025-08-15

**Authors:** Gabriela R. Mendeluk, Verónica A. Alonso

**Affiliations:** Departamento de Bioquímica Clínica, Hospital de Clínicas”José de San Martín”. Universidad de Buenos Aires. Instituto de Fisiopatología y Bioquímica Clínica- INFIBIOC, C.A.B.A, Argentina; Department of Clinical Biochemistry, Hospital de Clínicas”José de San Martín”. University of Buenos Aires. Institute of Physiopathology and Clinical Biochemistry- INFIBIOC, C.A.B.A, Argentina

**Keywords:** *BRAF V600E*, liquid biopsy, precision oncology, cancer, personalized treatment

## Abstract

**Introduction:**

The *BRAF V600E* mutation is one of the most relevant genetic mutations in precision oncology. This variation is commonly found in several types of tumors, including melanoma, colorectal cancer, and thyroid cancer, to name a few. Liquid biopsy has emerged as a minimally-invasive screening option for this mutation.

**Content:**

This review examines the molecular biology of the *BRAF V600E* mutation, its role in tumor progression, and its utility in the diagnosis, prognosis and treatment of specific types of tumors. In addition, the focus is also placed on liquid biopsy-based screening options and their advantages over solid biopsy.

**Summary:**

Detection of this mutation through liquid biopsy offers a less invasive alternative to conventional tissue biopsies, with potential utility in guiding treatment adjustments and monitoring therapeutic response.

**Outlook:**

The use of liquid biopsy for the detection of *BRAF V600E* is expected to widespread in clinical practice, leading to the optimization of disease monitoring and personalized treatments for cancer patients.

## Introduction

Precision oncology is defined as the detection of genetic variants that play a major role in oncogenesis to guide specific and effective treatments tailored to each patient [1]. This specialty is also used for the monitoring and prognosis of some particular types of tumors. Advancements in genomic analysis, the search for and identification of novel biomarkers, along with the development of new drugs and innovative clinical trial designs have established precision oncology as a cornerstone in the management of cancer [1], [2].

*BRAF V600E* is one of the most relevant driver mutations in many types of tumors. *BRAF V600E* is a mutation in the exon 15 of the *BRAF* gene, with a thymine-to-adenine change at nucleotide 1799 that leads to a valine to glutamic acid substitution at codon *600* [3].

*BRAF* (V-Raf murine sarcoma viral oncogene homolog B) is a *RAS*-regulated serine-threonine kinase acting as a driver in the mitogen-activated extracellular signal-regulated kinase-dependent signaling pathway *MAPK* (RAS- RAF- MEK- ERK). This pathway can be activated by the epidermal growth factor receptor (*EGFR*) and, under normal conditions, leads to cell proliferation. However, the occurrence of a gain-of-function mutation in oncoproteins such as *BRAF* favors the development of tumors [3]. It is worth noting that *BRAF* may harbor different types of mutations, categorized into class I, II, and III. *BRAF V600E* is the most frequent class I mutation in cancer, with *BRAF V600 K/D/R* mutations being of the same class but less frequent [4]. *Non-V600 BRAF* mutations are classified into class II and III [4].

Class II contains a mutation in the active segment of the kinase domain (*K601 E/N/T, L597V/Q/R*) and in the P-ring (*G464 V/E, G469 A/V/R*) [4].

Alike Class II, Class III is also characterized by a P-ring mutation, this time known as *G466 A/E/V*. This mutation may be also located in the catalytic ring (N581I/S/T) and the DGF motif of kinase domain (*D594 A/G/H/N, D596D/R*) [4].

There is a variety of other less frequent *BRAF* mutations not included in the I, II and III classification. These include kinase domain α-C-spiral short segment missing whole frame deletions or fusions with loss of the N terminus [4].

Class I *BRAF* mutations, specifically *BRAF V600E,* are strongly associated with tumors such as melanoma, colorectal cancer and thyroid cancer [4]. Class I *BRAF* mutations are frequently found in patients with non-*BRAF* dependent lung and colorectal cancer resistant to *EGFR* inhibitors. These types of tumors are sensitive to *BRAF* (*BRAFis*) and MEK inhibitors or a combination of the two [4].

Class II *BRAF* mutations are independent from RAS activity and are sensitive to *BRAFis*, *pan-RAF* inhibitors, *MEK* inhibitors or a combination of all [4]. In patients with non-small cell lung cancer (NSLC) with *EGFR* mutations, Class II *BRAF* mutations are considered to play a major role in acquired resistance to *EGFR* inhibitors [4].

Class III *BRAF* mutations are RAS-dependent. In the absence of concurrent *RAS* or *NF1* mutations, tumors are sensitive to tyrosine kinase receptor inhibitors and associated with a higher sensitivity to *EGFR* inhibitors. In colorectal cancer, these mutations are linked to higher survival post treatment, which distinguishes them from Class I *BRAF* mutations, associated with poor prognosis [4].

Other less frequent variants include *BRAF* gene deletions or fusions, which are not RAS-dependent and have a mechanism similar to that of Class II mutations [4].

A variety of studies suggest that MEK inhibition is an active approach in tumors with *BRAF* mutations outside the V600 locus, and in *BRAF* fusions. Thus, Subprotocol R of the NCI-MATCH of the National Cancer Institute tested the effectiveness of the *MEK* (trametinib) inhibitor in patients with solid tumors and lymphomas positive for *BRAFV600E*. Although trametinib did not show substantial activity in this population, newer agents to target *MAPK* signaling are in development, including ERK inhibitors, which have demonstrated moderate clinical activity in other studies [5].

Therefore, there is solid evidence that identifying the type of *BRAF* mutation is useful for prognosis and targeted therapy selection.

Although the other genetic mutations mentioned above have already been described, the literature has traditionally focused on and identified *BRAF V600E* as a key marker in the diagnosis, prognosis and monitoring of different types of tumors [3].

This literature review is aimed at uncovering the most relevant clinical applications of *BRAF V600E* detection through liquid biopsy in cancer patients.

We focused on the literature published on *BRAF V600E* in melanoma, papillary thyroid carcinoma, and colorectal cancer. These types of tumors were selected for the high prevalence, prognostic value, and availability of targeted therapies for the *BRAF V600E* mutation. This mutation has demonstrated clinical relevance, as its detection guides the selection of personalized therapies, thereby improving outcomes, and contributing to advances in precision oncology [4]. A detailed review of these types of tumors is provided below. Other tumors to which this mutation is relevant are also detailed.

## Melanoma

As many as 50 % of metastatic melanomas harbor *BRAF* point mutations, including *BRAF V600E* [6], [7]. *BRAFis* such as vemurafenib and dabrafenib are highly effective in advanced *BRAF V600E*-positive melanoma, with a response rate of 60–80 % [8]. These inhibitors are most frequently used in combination with *MEK* inhibitors (cobimetinib, trametinib, binimetinib) [6]. They are also used in adjuvant therapies following surgical resection to reduce the risk of relapse. The effectiveness of this approach was demonstrated in a phase 3 clinical trial involving patients who had underwent resection of a stage III *BRAF V600E* or *V600K*-positive melanoma followed by 12-month adjuvant therapy (dabrafenib + trametinib). Disease-free survival was observed to be higher in these patients [9]. A prospective, multicentric, phase 2 clinical trial was conducted to test a combination treatment with encorafenib (*BRAF*inhibitor) and binimetinib (MEK inhibitor) followed by radiotherapy in patients with *BRAF V600*-positive melanoma and brain metastases. The study showed a positive clinical effect in terms of intracraneal response rate, with an acceptable safety profile and a low incidence of serious adverse events [10].

The phase 3 DREAMseq clinical trial suggests the nivolumab/ipilimumab combination followed by a therapy with *BRAF* and *MEK* inhibitors (dabrafenib + trametinib) in case of progression [11].

## Thyroid cancer

Papillary thyroid carcinoma (PTC) is the most common histological type of differentiated thyroid carcinoma (75–85 % of cases) and is characterized by low mortality rates and good response to radioactive iodine therapy. Different types of thyroid cancers often harbor different genetic mutations. The development of PTC is closely related to somatic *BRAF* point mutations and rearrangements in the *RET/PTC1, RET/PTC3* and *NTRK1/3* genes. Interestingly, driver gene aberrations in well-differentiated thyroid cancer are mutually exclusive [12]. In the last decade, the *BRAF V600E* mutation has been reported to be prevalent in Western countries, ranging from 35 % in the USA to 60 % in Europe. In Asia, studies reveal a considerable between- and within-country heterogeneity in relation to the prevalence of this mutation in PTC [12].

The *BRAF V600E* gene mutation is associated with a higher risk of resistance to postsurgery radioactive iodine therapy [13]. A multicentric, randomized, open, phase 2 clinical trial revealed that the dabrafenib + trametinib combination was not superior in effectiveness to monotherapy with dabrafenib in patients with progressive *BRAF*-positive thyroid cancer refractory to radioactive iodine [14].

When the allele frequency of the mutation is increased in TPC, the tumor is more aggressive, larger in size, and with a higher number of lymph node metastases [15]. Based on the results of the ROAR clinical trial, the FDA approved the dabrafenib + trametinib combination for anaplastic thyroid cancer and advanced melanoma [16].

## Colorectal cancer

*BRAF V600E* mutations are found in around 10 % of patients with metastatic colorectal cancer, being an indicator of poor prognosis [17]. This finding predicts resistance to anti-EGFR monoclonal antibodies [17]. *BRAF V600E* mutations concurrent to an advanced age, a right colon primary tumor, and female gender are associated with poor response to chemotherapy and unfavorable prognosis. Moreover, these mutations are closely related to a low expression of mitmatch repair enzymes (MMR) and high microsatellite instability (*MSI-H*). Hence, molecular profiling is essential in all types of metastastic colorectal tumors, as this association has prognostic and therapeutic implications [18], [19].

In a prospective phase 3 study involving patients with metastatic MSI-H colorectal cancer, first-line treatment with a PD-1-targeted monoclonar antibody (pembrolizumab) showed a longer progression-free survival with fewer adverse events, as compared to the patients treated with chemotherapy [20].

S CAPSTAN CRC, a retrospective, observational European study examined the treatments available for *BRAF V600E*-positive metastatic colorectal cancer treated over a 4-year period. The treatment of choice was chemotherapy (FOLFOX: 5-FU, oxaliplatin and leucovorin) plus Anti-VEGF (bevacizumab). The *BRAF* inhibitor plus cetuximab was administered to only a few patients and as third-line therapy [21]. The results of a prospective phase 3 clinical trial, BEACON CRC, involving patients with metastatic colorectal cancer led to FDA’s approval of dual therapy with anti-EGFR and a *BRAF* inhibitor (cetuximab + encorafenib) [22]. The BREAKWATER clinical trial uncovered that the combination of dual targeted therapy (encorafenib and cetuximab) and chemotherapy in *BRAF V600E*-positive mCRC improve clinical outcomes when used as first-line therapy [22]. The combination of cytotoxic chemotherapy, which has a non-selective antitumor effect, and targeted therapy may overcome intratumor heterogeneity through an additive effect, targeting different cell populations, and ultimately improving clinical outcomes [22].

Even with this approach, response to treatments is still low in some patients. For this reason, other combinations have been tested, including a phase 2 clinical trial combining PD-1 (spartalizumab), *BRAF* (dabrafenib) and MEK (trametinib) inhibition, resulting in an augmented immune response, which is impaired in many types of tumors [8].

Table 1 shows the main clinical applications of *BRAF V600E* in clinical oncology in the three types of tumors selected.

**Table 1:** Primary clinical applications of detecting the *BRAF V600E* mutation in the most relevant solid tumors.

Type of solid tumor	Clinical applications	
	Prognosis	Targeted therapy/immunotherapy
Melanoma [6], [7], [8, 11]	Improved rate of response to targeted therapy in metastatic or unresectable melanoma.	In case of progression, *BRAF* inhibitors are recommended (ej: dabrafenib + trametinib) followed by immunotherapy (nivolumab + ipilimumab) are recommended.
Metastatic colorectal cancer [20], [21], [22]	Associated with poor prognosis	Combination of EGFR and *BRAF* inhibitors (e.g.: cetuximab + encorafenib). In case of MSI-H, immunotherapy is recommended (e.g.: pembrolizumab).
Thyroid cancer [13], [15], [16]	Associated with a higher risk for relapse and extrathyroidal extension in papillary thyroid carcinoma.	*BRAF* and MEK (dabrafenib + trametinib) inhibitors

Summary of the results of relevant studies and clinical trials on *BRAF V600E*.

## Other *BRAF V600E*-positive tumors

Non-small cell lung cancer (NSCLC) is a leading cause of mortality worldwide, rarely harboring *BRAF* mutations (0–3 %). In the few *BRAF*-positive cases reported, the use of oral *BRAF* inhibitors in combination with oral *MEK* inhibitors was reported to lead to higher response rates than standard therapy [23]. The detection of this mutation is associated with poor prognosis. For patients with metastatic disease, the combination therapy dabrafenib + trametinib is recommended [24]. *BRAF* fusions may also occur in NSCLC, although effective therapeutic approaches are not available in this case. A review was performed of the clinical characteristics, mechanism of action, and clinical management of the *BRAF* fusion to lay the foundations of the treatment of the *BRAF* fusion in NSCLC patients [24].

With regard to central nervous system tumors harboring this mutation, the therapy targeted against MAPK signaling pathway components was recently demonstrated to be effective [25]. The use of dabrafenib in combination with trametinib has been recently demonstrated to be effective in pediatric *BRAF V600* glyomam, which warrants future studies testing this combination as first-line treatment [26].

In relation to low-grade serous ovarian cancer, *BRAF* inhibitors as single agents were approved for the treatment of *BRAF*-mutated tumors. In some cases, the combination of *BRAF* inhibitors with *MEK* inhibitors delays the onset of resistance, as compared to the *BRAF* inhibitor single agent [27].

In the multicentric, phase 2 clinical trial, the combination of dabrafenib and trametinib demonstrated clinical tumor-agnostic activity in patients with rare *BRAFV600E* cancers, including anaplastic thyroid carcinoma, biliary tract cancer, gastrointestinal stromal tumor, small bowel adenocarcinoma, low-grade glioma, high-grade glioma, hairy cell leukemia, and multiple myeloma [28].

## Use of liquid biopsy to detect *BRAF V600E* in tumor circulating DNA

Liquid biopsy is an essential tool for detecting biomarkers in the bloodstream or in body fluids of cancer patients. This approach is based on the use of highly-sensitive molecular biology technologies such as qPCR (quantitative/real-time PCR) and NGS (next generation sequencing), which enable precise molecular profiling of tumor tissue [29].

Notably, cfDNA (cell-free DNA) is one of the most extensively studied analytes in liquid biopsy research. In general, cfDAN levels are higher in cancer patients than in healthy individuals and tend to increase as disease progresses and metastases appear; this is due to the massive release of genetic material from tumor cells [29]. However, high levels of cfDNA are not exclusive of tumors, as they may increase in pathological and non-pathological scenarios, such as after strenuous exercise or in postoperative periods. This makes it difficult to establish a direct association with cancer, as cfDNA is also found in healthy individuals and may have been released by non-tumor cells. Indeed, most of the circulating cfDNA is produced by non-malignant cells, having many of cell a germline cell origin [29], [30].

In contrast, ctDNA, which forms part of cfDNA, is tumor-specific, as it is derived from necrotic or apoptotic malignant cells and contains somatic tumor-specific mutations, thereby reflecting its dynamics [31], [32]. As ctDNA is not found in healthy people, it can be used to identify actionable mutations and monitor the course of cancer. Both, cfDNA and ctDNA, along with circulating tumor cells, are associated with tumor progression. Although cfDNA and ctDNA are found at low concentrations in peripheral blood, they are more likely to be detected than CTCs [32]. For this reason, the *BRAF V600E* mutation can be detected in ctDNA, as long as plasma samples have been stored at −80° for later analysis, or at −20° for immediate DNA processing [31], [32].

Following DNA extraction, quantification is performed. Spectrophotometry is generally used for routine determination of nucleic acid concentrations and purity. However, it is not DNA-selective, as it also detects other compounds such as RNA and proteins, resulting in an overestimation of DNA concentration [33]. For this reason, fluorimetry is the method of choice, involving the use of intercalating fluorophores that bind specifically to the nucleic acid under analysis [33].

## Detection techniques available

The total amount of ctDNA is only a minimal part of cfDNA. Such low concentrations can only be measured using highly-sensitive methods, particularly in early-stage tumors [34]. The most widely used techniques include quantitative/real-time PCR (qPCR), digital PCR (dPCR) and next generation sequencing (NGS) [34]. The dPCR technique is characterized by its high sensitivity. This technique splits the sample in thousands of individual reactions in wells containing 0, 1 or more DNA copies [35]. This way, rare but known variants, as well as subtle variations in copy number can be detected. This technique detects up to 1 mutated copy in 10,000, which corresponds to a MAF (minor allele frequency) of 0.01 %. In contrast, qPCR simultaneously analyze all signals, which may mask those with a lower intensity, with a MAF of up to 0.1 % [35], [36].

Another key characteristic of dPCR is its ability to perform absolute quantification of nucleic acids [35]. In contrast with qPCR, which enables relative quantification, dPCR reduces the probability of obtaining false positive results. In the context of liquid biopsy, dPCR minimizes the risk for false negative results, especially when the amount of DNA available is limited and mutations have a low frequency [35].

NGS detects MAF>1 % and has the advantage of screening for unknown variants, whereas qPCR or dPCR only analyze known variants [36].

## Advantages of liquid biopsy over solid biopsy for *BRAF V600E* determination

– **Non-invasive disease monitoring:** Liquid biopsy is used for the continuous monitoring of mutations such as *BRAF V600E* in blood samples, thereby preventing the need for repeat solid biopsies. This is especially useful in tumors located in anatomically inaccessible regions or in patients with advanced disease, in whom ctDNA analysis captures real-time changes and facilitates more agile therapeutic adjustments [37].
– **Minimal residual disease (MRD):** Liquid biopsy-based *BRAF V600E* detection allows for the early identification of persistent tumor cells after treatment [38]. As compared to solid biopsy, which only analyzes a static fragment of the tumor, liquid biopsy detects residual disease throughout the body. This enables more effective monitoring and early therapeutic interventions [38].
– **Tumor heterogeneity:** Liquid biopsy overcomes the limitations of solid biopsy, as it captures the genetic diversity of the entire tumor [39]. This technique identifies the co-occurrence of tumor subpopulations with and without the *BRAF V600E* mutation. This is crucial for evaluating resistance to targeted therapies and designing more precise and dynamic therapeutic approaches [39].

## Liquid biopsy validation and standardization

For liquid biopsy to be incorporated into clinical practice, it is essential that standard optimized analyte isolation protocols are established. To accelerate the development, validation, and clinical implementation of this technique, two public and private organizations in Europe and USA (BloodPAC) are gathering the experience of the industry and the Academy to ensure more robust and reproducible clinical trials [29].

## Commercial clinical trials on liquid biopsy-based *BRAF V600E* detection

There are several commercially-available kits for the detection of the *BRAF V600E* mutation based on *ctDNA* analysis. Some examples include:

– Guardant 360 (Guardant Health): A ctDNA panel that analyzes different types of tumors such as lung, and colorectal cancer, and melanoma, through NGS [40].
– FoundationOne Liquid (Foundation Medicine): A liquid biopsy panel that detects more than 300 cancer-related genes through NGS. This panel is useful for the treatment and detection of recurrent disease [40].
– Therascreen *BRAF V600E* Kit (QIAGEN): qPCR-based [41].
– EntroGen *BRAF* Mutation Analysis Kit: *qPCR*-based, designed to detect *BRAF V600E* [42].
– Idylla™ *BRAF* Mutation Test (Biocartis): qPCR-based, completely automated [43].

## Discussion

The *BRAF V600E* mutation is a genetic variant that plays a pivotal role in progression in different types of tumors. The relevance of this mutation in precision oncology has been widely demonstrated.

Although this review is focused on the detection of *BRAF V600E* in ctDNA through liquid biopsy, it is essential that evidence from tumor tissue-based studies is also considered. Current knowledge of the prognostic value of this mutation, as well as of the effectiveness of targeted therapies, is largely based on the results of clinical trials involving the use of tissue samples as the gold-standard diagnostic method. In our view, considering evidence from these studies is essential to better understand the clinical implications of detecting this mutation. Accordingly, we adopted an integrative approach that incorporates multiple data sources, using tissue biopsy data as a reference framework. This strategy facilitates the identification of both the strengths and limitations of employing this biomarker in liquid biopsy. Moreover, it proves particularly valuable in scenarios where tissue is inaccessible or when longitudinal monitoring of the disease is required.

This study is primarily focused on the relevance of this mutation in melanoma and thyroid and colon cancer, as it is a key driver mutation in these types of tumors. The utility of this mutation in NSCLC and other types of *BRAF V600E*-mutated tumors is also assessed.

Of all the forms of cancer considered in this study, this mutation has shown to be especially relevant in melanoma. The high frequency of this mutation in melanoma renders it the molecular target of choice in targeted therapies. Patients with advanced stage *BRAF V600*-positive cancer have a better prognosis, as this mutation improves the rate of response to personalized treatment.

In contrast with melanoma, the presence of this mutation in colorectal and thyroid cancer is associated with a poor diagnosis. It is in this context that clinical trials are conducted to test different combination therapies. In colon cancer, combinations of *EGFR, BRAF* inhibitors, chemotherapy, and immune therapy have been tested. In the case of thyroid cancer, *BRAF* and *MEK* inhibitors are under study.

The use of liquid biopsy for the detection of *BRAF V600E* in ctDNA will depend on the sensitivity required and on whether the mutated variant is known or not. Hence, dPCR would be indicated in early stage tumors upon suspicion of MRD or rare allelic variants. In the presence of unknown variants, NGS would be indicated. Although NGS is an expensive technique, costs will expectedly decrease and facilitate the collection of a higher amount of data, leading to a more efficient management of cancer [36].

Liquid biopsy-based detection of the *BRAF V600E* mutation offers a minimally-invasive method for monitoring the course of disease and assessing response to targeted treatments, emerging as a key tool in precision medicine. For the use of liquid biopsy to widespread in clinical practice, it is necessary that highly-sensitive technologies are available. However, some key elements remain to be solved, including the unavailability of appropriate indications for use, the limitations of the technique –such as its sensitivity–, the reporting of variants, the interpretation of results, its cost-effectiveness, and coverage by the Public Health System [29].

Liquid biopsy has different applications in oncology, being ctDNA the most widely used biomarker [29]. This technique has proven to be beneficial at different tumor stages, as it can serve as a diagnostic tool both at early and advanced metastatic stages [29]. Liquid biopsy is also used to complement tissue biopsies and other existing diagnostic and monitoring techniques. Liquid biopsy has facilitated cancer staging and monitoring, the detection of post-surgery minimal residual disease (MRD) in different types of tumors, and the monitoring of therapeutic response [29], [44]. Otherwise said, this technique facilitates the longitudinal monitoring of patients undergoing surgery or initial treatment, helps predict disease recurrence and provides therapeutic guidance. At disease onset, the patient starts with one single disease focus showing a distinctive molecular biology, but multiple metastases and distinct clones may emerge over time, giving rise to tumor heterogeneity. In contrast to tissue or solid biopsy, liquid biopsy reflects tumor heterogeneity [44], [45].

## Conclusions and future horizons

The evidence gathered in this thorough review of the literature identifies *BRAF V600E* as a key prognostic and follow-up biomarker, as well as a relevant therapeutic target.

The detection of this mutation in melanoma is a favorable prognostic marker, as targeted therapies such as *BRAF* and *MEK* inhibitors are known to significantly improve survival in carriers of this mutation.

In colorectal cancer, the presence of this mutation is related to a poor prognosis, being associated with resistance to conventional therapies and resulting in lower overall survival rates. Something similar occurs with thyroid papillary carcinoma, where the presence of this mutation is associated with more aggressive disease and a higher risk for relapse. However, in the two cases, its detection has utility in defining personalized therapeutic approaches.

In summary, screening for this mutation emerges as a useful tool for estimating the clinical course of the disease and defining therapeutic strategies in precision oncology, This is a diagnostic method for which preliminary results have been obtained from clinical trials; however, it has not yet been fully integrated into routine oncology practice. Detection through this technique offers several advantages over solid biopsy, including non-invasive monitoring of disease, detection of minimal residual disease, and a more comprehensive representation of tumor heterogeneity.

Future prospects include the standardization of operating protocols and the development of novel formulations targeting components of the signaling pathway under investigation, which may uncover previously unknown alternatives to improve patient survival and quality of life. In the future, *BRAF V600E* screening in healthcare centers might consolidate as a key tool in precision oncology, thereby contributing to optimizing the diagnosis and treatment of different types of tumors.

This study provides a deeper understanding of the clinical potential of liquid biopsy in detecting *BRAF V600E* in oncology.
